# Editorial: Community series in the role of toll-like receptors and their related signaling pathways in viral infection and inflammation, volume II

**DOI:** 10.3389/fimmu.2024.1505715

**Published:** 2024-10-18

**Authors:** Oliver Planz, Ralf Kircheis

**Affiliations:** ^1^ Department of Immunology, Faculty for Cell Biology, University of Tübingen, Tübingen, Germany; ^2^ Research & Development, Syntacoll GmbH, Saal an der Donau, Germany

**Keywords:** toll-like receptor, signaling pathway, viral infection, inflammation, tolerance

The second volume of our Community Series on “The Role of Toll-like Receptors and their Related Signaling Pathways in Viral Infection and Inflammation” delves into the intricate mechanisms through which TLRs influence the immune response against viral pathogens. This Research Topic, led by diverse researchers, expands our understanding of TLRs and their role in inflammation.

Included studies comprehensively examine how TLR activation regulates different stages of inflammation. For example, research by Butcher et al. highlights macrophage tolerance in TLR signaling, crucial for adapting to chronic endotoxin exposure and mitigating prolonged pro-inflammatory cytokine production. This adaptation aims to protect host tissues from damage, observed across various TLR ligands, particularly TLR4’s ligand lipopolysaccharide (LPS), which induces global shifts towards anti-inflammatory responses.

Additionally, Ricci-Azevedo et al. discuss lectins as TLR agonists with immunomodulatory properties. Plant lectin ArtinM and microbial lectins interact with TLR2 and TLR4, triggering NF-κB activation and IL-12 production *in vitro*. *In vivo* studies show these lectins confer resistance to intracellular pathogens. Lectins from pathogens like Toxoplasma gondii and Paracoccidioides brasiliensis also activate TLRs, suggesting potential for new pharmaceutical tools against infections and tumors.

Moreover, Lu et al.’s review underscores TLRs’ significant role in inflammatory bowel disease (IBD), a chronic global condition. Dysfunctions in TLR-mediated pathways contribute to IBD pathogenesis and influence treatment efficacy, prompting exploration of novel therapeutic strategies.

Lastly, Popotas et al. explore sex-specific immune responses in acute inflammatory diseases, attributing these differences to genes on the X chromosome encoding TLRs. Their review highlights distinct inflammatory patterns between sexes, crucial for tailoring clinical approaches.

This volume aims to advance our understanding of TLRs in inflammation, driving targeted therapy development and improving patient outcomes. We extend gratitude to all contributors for their invaluable insights and dedication to this critical field.

